# Fading Blue: Exploring the Causes of Locus Coeruleus Damage Across the Lifespan

**DOI:** 10.3390/antiox14030255

**Published:** 2025-02-22

**Authors:** Alessandro Galgani, Marco Scotto, Ugo Faraguna, Filippo S. Giorgi

**Affiliations:** 1 Department of Translational Research and of New Surgical and Medical Technologies, University of Pisa, 56125 Pisa, Italy; 2 Istituto Italiano di Tecnologia, 16163 Genova, Italy; 3 I.R.C.C.S. Stella Maris, Calambrone, 56128 Pisa, Italy

**Keywords:** locus coeruleus, noradrenaline, Alzheimer’s disease, neurodegeneration, Down’s syndrome

## Abstract

Locus Coeruleus (LC) is a brain nucleus that is involved in a variety of key functions (ranging from attention modulation to sleep–wake cycle regulation, to memory encoding); its proper function is necessary both during brain development and for brain integrity maintenance, and both at the microscale and macroscale level. Due to their specific intrinsic and extrinsic features, LC cells are considered particularly susceptible to damage concerning a variety of insults. This explains LC involvement in degenerative diseases not only in adults (in the context of neurodegenerative disease, mainly), but also in children (in relation to early hypoxic damage and Down’s Syndrome, among others). In this narrative review, we dissect the potential mechanisms through which LC is affected in different diseases, with a special emphasis on the high rate of activity it is subjected to and the oxidative stress associated with it. Further research aimed at deepening our understanding of these mechanisms is needed to enable the development of potential strategies in the future that could slow down LC degeneration in subjects predisposed to specific brain disorders.

## 1. Introduction

The subcortical nucleus LC has been the object of extensive research in recent decades not only in terms of its physiological role (noteworthy, it represents the anatomical substrate of the “ascending reticular activating system” described by Moruzzi and Magoun 75 years ago [1], and it has been shown to play key roles in several cognitive functions, the sleep/wake cycle, and in modulating several cell mechanisms) but also as a brain nucleus degenerated in a variety of brain diseases. The loss of noradrenaline (NA) or its co-transmitters in its target structures, or the recently hypothesized spreading of toxic substances through its degenerating axons, might represent an early, pathogenetically relevant event in neurodegenerative diseases (NDDs). In this narrative review, we will report its functional anatomical features and a brief overview of the main NDDs in which LC is impaired, with the aim to introduce the potential mechanisms which make LC particularly vulnerable. The latter mechanisms have been extrapolated from the literature published so far in PubMed. An insight into the mechanisms through which LC degeneration occurs in different NDDs is key for the development of potential strategies aiming to prevent or at least reduce/slow down the LC degeneration in subjects predisposed to specific brain disorders.

## 2. The Locus Coeruleus: Ontogenesis and Functional Anatomy

The LC belongs to the isodendritic core of the brainstem, a group of phylogenetically ancient nuclei characterized by a widespread and diffuse efferent system and a significant convergence of afferent inputs [2].

The development of LC is an early step in human neurogenesis, and its differentiation is crucial also for the physiological ontogenesis of the whole central nervous system (CNS). Tyrosine hydroxylase (TH)- and dopamine-β-hydroxylase (DBH)- positive cells appear within the rhombomere 1 of the developing hindbrain already at the fifth gestational week [3,4,5]. The differentiation of NA cells coincides with the development and differentiation of their vascularization. During this phase, capillaries already exhibit well-formed walls and the characteristic features of an organized blood–brain barrier (BBB) [6]. A complex molecular interplay orchestrates this developmental stage [7,8,9,10]; bone morphogenetic proteins 2,5 and 7, fibroblast growth factor 8, and Wnt proteins promote the expression of TH gene in LC developing cells [8], while the transcriptional factors Phox2a and Phox2b induce the expression of DBH and NA Transporter (NAT) genes [10,11].

Noradrenergic innervation starts to spread onto the cortical plate within the thirteenth gestational week, following a rostro-caudal trajectory, from frontal regions to occipital ones [12]. Since then, a bidirectional interaction is established between LC and the NA innervated areas, with the former promoting the differentiation of the latter and vice versa [13,14]. Indeed, animal studies suggest that NA might stimulate the development of the CNS from its earliest stages, by promoting the differentiation of neural progenitors from ectodermal cells [15,16]. Later on, NA fibers play key roles in the regulation of forebrain and spinal cord structural organization [17,18,19,20,21,22], particularly promoting the correct differentiation of the cortex and its layer architecture [21].

LC-NA cell population continues to grow also after birth, progressively separating into two clusters, one rostral and one caudal, probably reflecting the different CNS area they project to [23]. In line with this, NA innervation expands massively, rapidly reaching the final architecture observed in adulthood [24]. It is worth noting that also after birth, NA still exerts modulatory effects on neuronal development [17], promoting synaptogenesis [18]—crucial in the earliest post-natal neural development—and modulating hippocampal neurogenesis [25,26,27,28,29].

In the adult, LC is a small, tube-shaped nucleus located just below the floor of the fourth ventricle [30,31]. Its most characteristic morphological feature is the dark blue/black color, which is due to the accumulation within the NA neurons of neuromelanin (NM), a catabolic by-product of NA itself [32]. As said, the LC provides a wide and diffuse projection system, virtually reaching the whole CNS [33,34]. However, the connections established by the LC probably constitute segregated neural circuits, allowing the nucleus to modulate specifically the activity and functions of target areas [35,36]. LC neurons can release NA in two fashions: classically as a neurotransmitter, at the level of the synaptic cleft, and as a paracrine hormone, through varicosities emerging all along their unmyelinated axons [37,38]. Such a dual modality reflects the double level at which LC-NA system works, both as a regulator of neural networks and as a modulator of CNS homeostasis [39,40,41,42,43,44,45,46,47,48,49]. The LC plays a key role in the regulation of vegetative life and the autonomic nervous system; through its connection with other nuclei of the ascending activating system [1] and the hypothalamus [41], the LC is a wake-promoting nucleus and takes part in the regulation of circadian rhythms [41]. In the hippocampus, LC-NA fibers promote memory encoding and formation [44,47,50,51]. LC projections to the cortical sensory area increase the signal-to-noise ratio and allow focusing on salient stimuli [39,52] while targeting the frontal cortices; it is a crucial part of the attention networks [45,53,54]. Finally, at the cellular level, NA exerts many housekeeping roles, modulating microglia activity [43] and regulating neurovascular coupling [55].

## 3. The Involvement of Locus Coeruleus in Human Disorders at Different Ages

The LC is involved in a variety of diseases across different age groups. Most of the evidence focuses on the degeneration or alteration of this nucleus in NDDs affecting adults and the elderly. In particular, since the early 1980s, studies have shown significant loss of LC neurons in patients with Parkinson’s disease (PD) and Alzheimer’s disease (AD) when compared to age-matched controls [56,57,58,59,60].

These early studies were primarily based on qualitative brain specimen analyses. More recently, unbiased stereological techniques have confirmed LC neuronal loss [61,62].

Concerning NA levels in PD patients, in his early studies, Hornykiewicz (the father of the neurochemistry of PD) and colleagues demonstrated that the degeneration of LC neurons in PD patients was significant and even more pronounced than that of dopaminergic (DA) neurons in the substantia nigra pars compacta (SNpc). Remarkably, in various brain regions, no overlap in NA levels was observed between PD patients and healthy controls, with the former consistently showing lower concentrations. Moreover, these non-overlapping differences were observed in more regions for NA than for DA [63,64,65,66].

More recently, the involvement of the LC has been repeatedly documented as occurring in the early stages of PD (for a review, see [59]). A seminal paper by Braak et al. (2003) is particularly noteworthy. It showed that the accumulation of alpha-synuclein (α-syn) and Lewy bodies within the LC occurs years before these changes appear in the SNpc [67]. This observation aligned with the hypothesis that the early degeneration of the LC may contribute to, or even cause, the degeneration of SNpc DA neurons. This hypothesis has also been supported by experimental studies dating back to the early 90s. The first of those was performed by Colpaert’s group in monkeys and afterward was confirmed by a variety of studies in rodents [68]. All of those reports showed that a previous lesion of the LC accelerates the degeneration of SNpc DA neurons when exposed to specific neurotoxins, primarily MPTP and substituted amphetamines [69,70,71].

The involvement of the LC in diseases in which α-syn accumulation plays a critical role has been confirmed in the past three decades concerning disorders other than PD. In particular, it has been shown that LC impairment is significantly involved also in multiple system atrophy (MSA) and Lewy Body Dementia (LBD) [72,73]. Even more interestingly, in the last two decades, several studies have shown that the LC is involved early in idiopathic REM sleep behavior disorder, which is mainly due to α-syn accumulation and is well known to precede the onset of both motor and non-motor signs and symptoms of PD [74,75,76,77,78].

Regarding other NDDs of the elderly in which severe LC degeneration occurs, the one that has been studied in the most detail is, by far, Alzheimer’s disease (AD). The first *post-mortem* studies documenting LC degeneration in AD patients date back to the 1970s [57,58,79,80,81,82,83,84]. In the following decades, more evidence accumulated confirming this finding, culminating in the most comprehensive anatomopathological analysis of LC features in AD conducted by Kelly et al. in 2017 [61]. This latter study showed, for the first time using unbiased stereological analysis, that LC degeneration is already well established with significant neuronal loss in patients with mild cognitive impairment (MCI). Interestingly, a few years before Kelly et al.’s study, Braak’s group had already shown that decades before the onset of the first clinical signs of AD, there is already an accumulation of abnormal proteins—specifically hyperphosphorylated tau protein (pTau)—in the cell bodies and later in the axon terminals of LC neurons [85]. This protein accumulation is a precursor to neurofibrillary tangles (NFTs) formation in AD. This led to the hypothesis that the first signs of tau pathology in AD patients may occur at the level of the LC, indeed, and only later spread to other structures. Based on their observations, Braak and Braak proposed a modification to the tau pathology staging classification, adding stages 1a, b, and c to describe the progressive accumulation of pTau starting from the LC cell body, extending through the axon and up to the terminals [85]. They even postulated that the early accumulation in the cell body and axon terminals could later lead to the spreading of tau pathology to LC axon target regions. Specifically, they proposed that the brain regions more densely innervated by LC axon terminals, such as the entorhinal cortex and other limbic structures, are the first cortical areas to accumulate tau pathology, potentially spreading from LC axon terminals to those cortical regions [86]. This hypothesis has been partially confirmed in recent experimental models [87,88].

Interestingly, LC involvement has also been observed in NDDs affecting younger individuals. Down’s syndrome, which is associated with an increased accumulation of amyloid in the brain and early brain degenerative phenomena resembling those occurring in AD, has been shown to involve LC degeneration starting at a young age [89,90]. This is particularly interesting since these patients primarily experience amyloid-related pathology [91]. In children, LC alterations have also been shown in other disorders. Specifically, *post-mortem* studies in subjects affected by perinatal hypoxic–ischemic insults have revealed LC alterations [6,92,93,94]. This can be interpreted as further evidence of LC vulnerability to various insults. Details on the potential mechanisms making the LC particularly susceptible to a variety of insults are provided in the following paragraph. This section may be divided by subheadings. It should provide a concise and precise description of the experimental results, their interpretation, as well as the experimental conclusions that can be drawn.

## 4. The Main Features of LC That May Underlie Its Susceptibility to Damage

**Figure 1:**
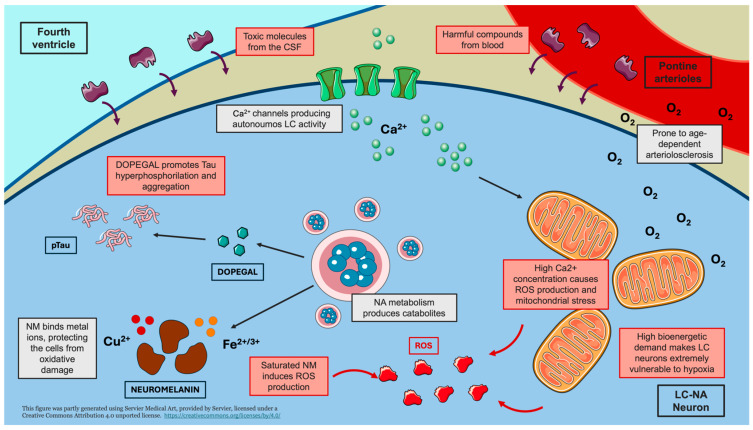
Mechanisms underlying LC vulnerability. The schematic representation highlights the main mechanisms contributing to the vulnerability of LC neurons to damage. The intrinsically high firing activity and bioenergetic demand of LC-NA neurons lead to mitochondrial dysfunction, oxidative stress, and increased susceptibility to hypoxic damage. The anatomical proximity of the LC to the fourth ventricle and its abundant capillary network expose LC neurons to circulating toxic compounds. Furthermore, the catabolic by-product of noradrenaline, DOPEGAL, promotes tau hyperphosphorylation (pTau) and aggregation. Limited neuromelanin storage may result in the release of metal ions, inducing the production of reactive oxygen species (ROS). Creating using Servier Medical Art with minor modifications under a CC BY 4.0 license.

In the present narrative review, we describe the main potential mechanisms emerging from the literature on LC neurodegeneration (Figure 1).

As described in the previous paragraph, the LC is affected in a variety of neurodevelopmental and neurodegenerative diseases [59,95,96,97]. If, on the one hand, this is not surprising considering the plethora of neural functions and networks in which it is involved, its remarkable vulnerability to damage, on the other hand, appears to be quite peculiar. The reasons underlying this characteristic, even though far from being clearly understood, are probably related to the LC’s very nature, being it a pontine monoaminergic nucleus with a strong and long-lasting firing activity [98,99].

Indeed, LC fires tonically during wake, with phasic activity appearing following unexpected stimuli or during tasks requiring focused attention [39]. Even though their activity is reduced in NREM sleep, LC neurons are completely silent only during REM phases [49]. Because of this, LC has an extremely high bioenergetic demand, whose homeostatic fulfillment seems to depend on the proper functioning of mitochondria [100,101]. However, in the LC, mitochondria seem to be subjected to prolonged and frequent oxidative stress peaks, due to the continuous and reiterate influx of Ca^2+^ [100]. The latter enters the mitochondria through the calcium channels that produce the spontaneous tonic firing of LC neurons, which is, as said, particularly high [100]. Thus, such elevated LC activity by itself might be the cause of progressive damage to mitochondria, hampering the energetic supply of NA neurons [98]. In line with this, it is not surprising that LC is also particularly vulnerable to hypoxic damage [102]. Neuropathological studies performed on children who died from perinatal hypoxia showed dramatical damage to the LC [6,92,93].

The second element that should be considered is the topographical location of the LC, just beneath the floor of the fourth ventricle [31]. LC-NA neurons are thus particularly exposed to possible toxins and harmful compounds circulating in the cerebrospinal fluid (CSF) [103]. Noteworthy, some pieces of evidence suggest that LC neurons might also be particularly sensitive to peripheral chronic inflammation, whose by-products might reach the nucleus through the CSF or the bloodstream [104]. Regarding blood supply, the LC is further disadvantaged by its poor vascularization. In fact, the perfusion of this nucleus is granted by perforating pontine arterioles, which are particularly prone to age-dependent arteriolosclerosis [105]. At the microscopic level, LC-NA neurons have a very close relation with capillaries [106], which are particularly abundant due to the chemoreceptive roles of the nucleus [98] and are, thus, exposed to circulating toxins and viruses [107,108].

Beyond being exposed to exotoxins, some hypotheses suggest that the LC itself might produce harmful compounds [109]. As said above, one of the most iconic features of LC is the production of NM during the catabolism of NA [32,110]. NM binds iron and copper ions, which are crucial for neuronal metabolism, protecting NA cells from oxidative damage [111]. However, the capability of LC neurons to store NM-metal ions compounds might be overcome with aging, producing possible detrimental consequences [111]. Due to the loss of NM’s ability to bind metal ions, the latter might induce the production of ROS, which, in turn, might cause cell death through apoptosis [111]. Then, dead neurons might release NM granules, which could activate nearby microglial cells and trigger neuroinflammatory processes, which might increase cell stress within other NM-containing neurons, leading to their death and a vicious cycle [112].

Finally, a huge number of studies highlight the sensitivity of LC to the accumulation of aberrant proteins, particularly pTau [98,99,103]. Indeed, according to Braak’s revised tau pathological staging (already described above), the LC might be the first brain structure affected by AD pathology [85,86]. While pTau formation and accumulation might be facilitated and exacerbated by all the mechanisms listed above [98,99,103], current evidence suggests that there might also be a more specific cause for its accumulation within LC neurons, related to NA metabolism itself [113,114,115]. In vitro studies showed that 3,4-dihydroxyphenylglycolaldehyde (DOPEGAL), another byproduct of NA, might trigger the activation of an arginine-peptidase, which cleaves tau protein in an isoform more prone to hyperphosphorylation [115], promoting its aggregation and propagation through LC axons to the other brain regions [114]. It is worth noting that recent very elegant studies suggested that this process might be exacerbated by sleep disturbances and deprivation [116], the latter being a mechanism responsible, by itself, for LC neuron degeneration through disruption of cellular metabolism [117].

## 5. Oxidative Stress as a Common Mechanism of LC Degeneration

Intriguingly, most of the pathological pathways reported in the previous paragraph lead to the production of ROS [98,99]. Oxidative damage has been identified as critical for vulnerability, affecting protein metabolisms and mitochondria functioning in neurons [118,119]. Globally, there are two main reasons for which LC suffers from oxidative stress, and they are its high activity rate and the NA metabolism [100,111]. As described above, LC autonomous activity is driven by voltage-dependent Ca^2+^ channels, which allow a continuous flow of Ca^2+^ within LC-NA neurons cytosol [120]. Ca^2+^ then enters mitochondria causing the activation of nitric oxide synthetase (NOS) and the consequent release of NO [100]. NO is likely to slow down the reaction of oxidative phosphorylation, reduce the mitochondrial membrane potential, and lower the Ca^2+^ influx, in order to avoid the activation of the apoptotic process [100]. As a side effect, superoxide and other ROS are produced, damaging the mitochondria themselves, other organelles, and cytosolic proteins [100]. This same mechanism might be elicited by hypoxia, a condition to which LC is very sensitive [92,102]. Indeed, studies performed on children who died of perinatal hypoxia showed a dramatic involvement of this nucleus, as revealed by severe depigmentation and reduction in cell counts [6,92,93]. In hypoxic conditions, the insufficient O_2_ supply to LC neurons might hamper Na^+^/K^+^ pump functioning, causing the reduction in the transmembrane potential and allowing an abnormal influx of Ca^2+^ ions [118], inducing the same mitochondrial alterations described above [100,120]. Even though this latter mechanism might occur indistinctly in other CNS areas as well, the particularly high bioenergetic demand of LC, evidenced by its abundant capillary bed [106] and elevated firing activity [39], might make it a relatively specific target of hypoxic-oxidative damage.

On the other hand, NA metabolism exposes LC neurons to persistent oxidative stress [121]. Firstly, the entire enzymatic machinery needed for NA synthesis requires metal ions, namely iron and copper [121]. NA is produced starting from the amino acid phenylalanine, which is hydroxylated into tyrosine by phenylalanine hydroxylase through an iron-mediated reaction [122]. Tyrosine is then hydroxylated by TH in another iron-mediated reaction, being converted to L-DOPA [123]. L-DOPA is then decarboxylated by DOPA decarboxylase, obtaining DA [124], which is then hydroxylated into NA in a copper-mediated reaction by DBH [125]. The turnover of those enzymes causes a continuous movement of iron and copper within NA cells, which is partly bound and chelated by NM [32], causing the production of ROS and the direct oxidation of proteins and DNA [111]. Moreover, hydroxylation reactions produce quinone species as by-products, the most harmful of which might be DA-quinone [121]. DA-quinone shows high reactivity with other molecules, inducing oxidation of protein and lipids and propagating oxidative damage through the production of ROS [126]. It is worth noting that DA-quinone might be one of the most relevant substrates leading to the production of NM itself [121], further highlighting the complexity of the anti-oxidative defenses of LC-NA neurons, and, at the same time, how strongly exposed these cells are to oxidative damage. In this context, the hyperphosphorylation of the tau protein induced by DOPEGAL might jeopardize NA vesicle organization and storing [113,115,127]. This might prevent the uptake of DA into vesicles, where DBH is localized [125], increasing its concentration in the cytosol and increasing the risk of its oxidation into DA-quinone [121].

NA degradation might also cause oxidative stress. Monoamine-oxidase (MAO), the enzyme responsible for the first step of NA catabolism, increases the production of oxidative compounds [128,129]. The resulting product is DOPEGAL [130]. DOPEGAL is a well-known neurotoxic compound (when its production overwhelms the activity of cellular enzyme aldehyde-dehydrogenase), inducing the formation of free radicals and ROS [131] and affecting mitochondria, both increasing their permeability to Ca^2+^ and acting as a second messenger for apoptosis activation [131,132,133].

**Figure 2:**
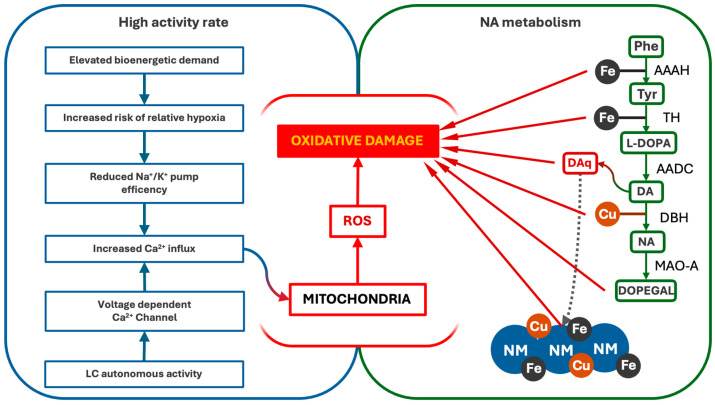
Pathways of oxidative stress and damage in LC-NA neurons. LC’s high firing activity and NA metabolism contribute to persistent oxidative stress. The autonomous activity of LC drives Ca^2+^ influx, which interferes with oxidative phosphorylation and promotes ROS formation, damaging the mitochondria and other cellular components. Additionally, NA synthesis and degradation generate oxidative by-products, including DA-quinones (DAq) and DOPEGAL, further increasing ROS levels and mitochondrial vulnerability. AAAH (Phenylalanyl Hydroxylase), TH (Tyrosine Hydroxylase), AADC (L-DOPA decarboxylase), DBH (dopamine-β-Hydroxylase), MAO-A (Monoaminoxidase A).

Altogether, these pieces of evidence underline how LC-NA neurons are particularly affected by oxidative stress, which results from both the alteration of mitochondrial integrity and the metabolism of NA (Figure 2).

## 6. Consequences of LC Dysfunction and Degeneration

The specific focus given to LC involvement in neuropsychiatric disorders is not pointless, considering the wide range of neural and cellular activities that are under its regulation and modulation. LC dysfunction can occur both as pathological hyperactivity [95,134,135] and as a degenerative loss of function [59,136]. In light of what was reported in the two previous paragraphs, it might not be considered too speculative to associate the two conditions as LC hyperactivity is linked to NA neurons’ progressive damage [98,100].

In neurodevelopmental disorders (NdDs), such as autism or attention-deficit hyperactivity disorder (ADHD), an abnormal hyperactivity of LC has been observed and linked to a deficit of attention [53]. LC firing can be categorized into two distinct modes: tonic and phasic discharge [137]. Tonic activity is primarily influenced by the state of vigilance (e.g., sleep vs. wakefulness) and external environmental conditions (e.g., calm vs. stressful situations). In contrast, phasic activity is triggered by novel or unexpected stimuli, as well as cognitive tasks that require sustained attention [39,40,137]. Disruptions in this delicate balance are likely to be at the core of attentional dysfunctions observed in NdDs. In affected individuals, LC exhibits basal hyperactivity, characterized by an increased frequency of tonic firing which impairs phasic responses. This dysregulation compromises both attentional focus and flexibility, ultimately leading to deficits in cognitive performance.

Similarly, the abnormal firing of the LC has been associated with sleep disturbances [48,49]. Human studies performed through LC-MRI and fMRI showed how higher LC activity during wake time is associated with poorer REM sleep quality [138]. Furthermore, a specific degeneration of LC was found in patients suffering from REM behavioral sleep disorder (RBD) [139,140,141], a prodromal condition to PD and other synucleinopathies [142]. As a possible explanation, the loss of inhibitory control on LC has been proposed [139,140]. During sleep, LC is inhibited by GABAergic projection coming from the hypothalamus and the pericoerulear area [41]. Current evidence suggests that impairment of the dendritic arborization of the LC-NA neurons, due to the ongoing degenerative disease, might reduce the sensitivity to inhibitory projections, increasing LC wake-time activity and impairing its silencing during sleep [49,138,140]. In this context, the consequences of LC impairment may extend far beyond a reduction in sleep quality. Indeed, very recent studies suggest that LC infraslow activity during NREM sleep may play a crucial role in preserving healthy sleep microarchitecture and regulating glymphatic system flow [143,144]. The glymphatic system is a relatively new and increasingly affirmed concept that describes a drainage system specific to the brain parenchyma. It relies on the communication between interstitial fluid and CSF through the perivascular spaces surrounding blood vessels entering the CNS from the meninges [145,146]. Functioning similarly to the lymphatic system of the body, it facilitates the clearance of waste compounds and toxic proteins, such as amyloid and tau [145,146]. Additionally, it is likely to play an essential role in the CNS immune response by promoting antigen presentation [147] and peripheral immune cell recruitment [148,149]. The flow of CSF within glymphatic system is thought to be driven by physiological arteriolar constriction and dilation alternation, a process that is particularly prominent during NREM sleep [144,145]. Intriguingly, experimental evidence suggests that LC-NA may regulate this vasomotion, thereby ensuring proper glymphatic system function [144]. Consequently, LC impairment could compromise CSF flow, leading to the accumulation of harmful compounds, as observed in an AD animal model for amyloid protein accumulation [150] or in a Traumatic Brain Injury model for the tau protein [151].

LC-NA system modulates also learning and memory, with a specific effect on emotional-salient and novelty-related events [44,152,153]. Experimental data shows that LC-NA projections promote memory encoding by inducing CA1 pyramidal cell activation [154], regulating long-term depotentiation [47] and preserving hippocampal neurogenesis [26]. In line with this, human studies performed in AD patients showed an association between LC integrity and memory performances, pointing out how the progressive degeneration of LC occurring in AD was associated with the level of cognitive impairment [153,155,156,157].

As already reported, LC impairment does not bear only behavioral consequences. Considering the crucial roles NA plays in maintaining brain homeostasis, the loss of LC modulation was associated with many detrimental effects at the cellular level [42,43,96,158]. NA regulates microglia, keeping it in the quiescent state M2 and exerting an anti-inflammatory effect [158]. Experimental lesions of LC in AD animal models caused the increase in pro-inflammatory cytokines release [159,160,161], the exacerbation of microglial activation [162,163], and the impairment of the phagocytosis-mediated amyloid clearance [164]. At the same time, NA depletion has been associated with BBB disruption at the brain capillary level [165]. The loss of integrity and the increased permeability that follows might lead to the passage of plasmatic proteins and compounds into the interstitial space, causing the activation or the exacerbation of neuroinflammation [166,167]. Moreover, the LC-NA system also plays a role in modulating neurovascular coupling, ensuring the fulfillment of energetic demands by inducing vasoconstriction in brain regions surrounding the activated areas, thereby focusing blood flow to the latter [168]. The impairment of this regulation might impair neurovascular coupling, causing regional hypoxia and leading to ischemic damage [42]. Finally, as NA promotes the transcription of surviving factors and regulates the expression of proteins needed for proper neuronal functioning [169,170], the impairment of LC might affect brain homeostasis also through this molecular mechanism.

## 7. Conclusions and Future Directions

The intrinsic features of the LC predispose it to neurodegeneration. The loss of its axon terminals leads to a loss of neuroprotective effects by norepinephrine (NA) or its co-transmitters on target neurons [169]; alternatively (or concomitantly), before the onset of degeneration of LC axon terminals, these may represent the preferential route through which toxic proteins could spread to target regions [86]. In the context of personalized medicine, early replacement of adrenergic and/or peptidergic (as neuropeptides are NA co-transmitters) receptor stimulation in LC target neurons could be particularly beneficial for patients in whom alterations in LC features can be promptly demonstrated in vivo. In this regard, it is worth noting the strong potential contribution of specific LC-sensitive MRI sequences that have been recently set up in patients [171,172]. Similarly, early documentation of altered NA and/or metabolites (including DOPEGAL) levels in the CSF could help identify patients at risk for early LC deterioration. More recently, other proxies, such as the pupil dilation pattern, have been linked to LC activity. Thus, in selected patients potentially vulnerable to a reduction in LC neuroprotective effects, specific drugs that enhance NA tone might be used. In line with this, recent studies have explored potential treatments, such as the CNS NAT inhibitor atomoxetine [173]. The more accessible these early signs of LC alteration become through detailed (likely multi-parametric) in vivo analysis, the more feasible the development of early LC-related treatments will be, hopefully leading to disease-modifying (and individualized) interventions. These treatments could be applied not only to elderly patients who are potentially at risk for neurodegenerative diseases but also to children who experience early insults that may be associated with LC degeneration. In such cases, the use of LC-potentiating approaches during specific time intervals after such insults could even enable prophylactic treatments for LC degeneration.

## Abbreviations

The following abbreviations are used in this manuscript:

AD	Alzheimer’s Disease
BBB	Blood–Brain Barrier
CNS	Central Nervous System
CSF	Cerebrospinal Fluid
DA	Dopaminergic
DBH	Dopamine-β-hydroxylase
DOPEGAL	3,4-Dihydroxyphenylglycolaldehyde
LBD	Lewy Body Dementia
LC	Locus Coeruleus
MCI	Mild Cognitive Impairment
MSA	Multiple System Atrophy
NA	Noradrenaline
NAT	Noradrenaline Transporter
NDD_s_	Neurodegenerative Diseases
NFT_s_	Neurofibrillary Tangles
NM	Neuromelanin
PD	Parkinson’s Disease
ROS	Reactive Oxygen Species
SNpc	Substantia Nigra pars compacta
TH	Tyrosine Hydroxylase
